# An innovative application of time-domain spectroscopy on localized surface plasmon resonance sensing

**DOI:** 10.1038/srep44555

**Published:** 2017-03-10

**Authors:** Meng-Chi Li, Ying-Feng Chang, Huai-Yi Wang, Yu-Xen Lin, Chien-Cheng Kuo, Ja-an Annie Ho, Cheng-Chung Lee, Li-Chen Su

**Affiliations:** 1 Thin Film Technology Center/Department of Optics and Photonics, National Central University, Taoyuan 32001, Taiwan; 2 BioAnalytical Chemistry and Nanobiomedicine Laboratory, Department of Biochemical Science and Technology, National Taiwan University, Taipei 10617, Taiwan; 3 Department of Optoelectric Physics, Chinese Culture University, Taipei 11114, Taiwan

## Abstract

White-light scanning interferometry (WLSI) is often used to study the surface profiles and properties of thin films because the strength of the technique lies in its ability to provide fast and high resolution measurements. An innovative attempt is made in this paper to apply WLSI as a time-domain spectroscopic system for localized surface plasmon resonance (LSPR) sensing. A WLSI-based spectrometer is constructed with a breadboard of WLSI in combination with a spectral centroid algorithm for noise reduction and performance improvement. Experimentally, the WLSI-based spectrometer exhibits a limit of detection (LOD) of 1.2 × 10^−3^ refractive index units (RIU), which is better than that obtained with a conventional UV-Vis spectrometer, by resolving the LSPR peak shift. Finally, the bio-applicability of the proposed spectrometer was investigated using the rs242557 tau gene, an Alzheimer’s and Parkinson’s disease biomarker. The LOD was calculated as 15 pM. These results demonstrate that the proposed WLSI-based spectrometer could become a sensitive time-domain spectroscopic biosensing platform.

White-light scanning interferometry (WLSI) is an effective optical measurement system for surface topography in the optoelectronic and semiconductor industries and the strength of the technique lies in its ability to provide fast and high resolution measurements. Recently, it has been extended to applications in surface and thickness measurements of thin films and transparent films^1^. Briefly, the principle of WLSI is based on the production of low coherence correlograms (temporal interferograms) when the optical path difference between an object and reference beams in a Michelson or Mirau interferometer approaches zero^2^,^3^,^4^,^5^. Then, the amplitude and phase in the frequency domain can be obtained by a Fourier transform of the temporal interferograms. Finally, the surface profile and properties of thin films, such as the thickness and refractive index, can be retrieved by either the Fourier amplitude or phase^1^,^6^. WLSI can also be used as a spectrometer because a time-domain signal can be converted to the frequency domain using the Fourier transform. In addition, a WLSI-based spectrometer can become a hyperspectral imaging system that is able to measure a whole image and spectrum at the same time without wavelength scanning and spatial scanning^7^,^8^. However, the spectral resolution of WLSI is restricted by the scanning distance of the moving mirror when WLSI is used as a spectrometer. Typically, the traveling range of the moving mirror is limited to the micrometer scale^9^,^10^,^11^, corresponding to a spectral resolution of approximately 10 nanometers in the visible spectrum. Unfortunately, such a spectral resolution is far worse than that of commercial spectrometers. To the best of our knowledge, the use of WLSI as a spectrometer has not been reported to date.

The most commonly used method for spectral analysis in chemistry and biology is the measurement of a spectral peak position or a corresponding intensity value^12^,^13^,^14^,^15^. As with any traditional spectrometer, performance is highly affected by the spectral resolution. An alternative method of spectral analysis, which has been proposed in surface plasmon resonance (SPR) systems, is based on calculating the spectral centroid, the geometrical center of a spectrum^16^. The centroid algorithm is anticipated to greatly reduce noise compared to direct estimations of the spectral peak position because more data points (wavelengths) are considered in the calculation^17^. We believe that such an algorithm could significantly improve the performance of any spectrometer with poor spectral resolution.

In this study, we integrated the spectral centroid algorithm with a breadboard of WLSI as a time-domain spectroscopic system. In addition, the performance of the WLSI-based spectrometer was tested and compared with a conventional UV-Vis spectrometer by measuring the extinction spectrum shift of localized surface plasmon resonance (LSPR) related to the refractive index variation. Furthermore, a WLSI-based spectrometer was implemented to measure the LSPR extinction spectrum shift in an oligonucleotide-induced gold nanoparticle (GNP) aggregation bioassay. To develop a sensitive LSPR-based detection assay, gold nanourchins (GNUs) were chosen since their spiky uneven surfaces cause a larger redshift in the LSPR extinction spectrum compared to that of spherical particles^18^. Experimentally, the rs242557 tau gene was chosen as a model analyte. Recent studies have identified that the rs242557 is a key regulatory polymorphism influencing microtubule associated protein tau (MAPT) expression, which is the risk factor for developing Alzheimer’s and Parkinson’s diseases^19^,^20^. The limit of detection (LOD) of the proposed system for rs242557 tau gene detection is 15 pM.

## Results

### Accuracy analysis of the WLSI-based spectrometer

**Figure 1:**
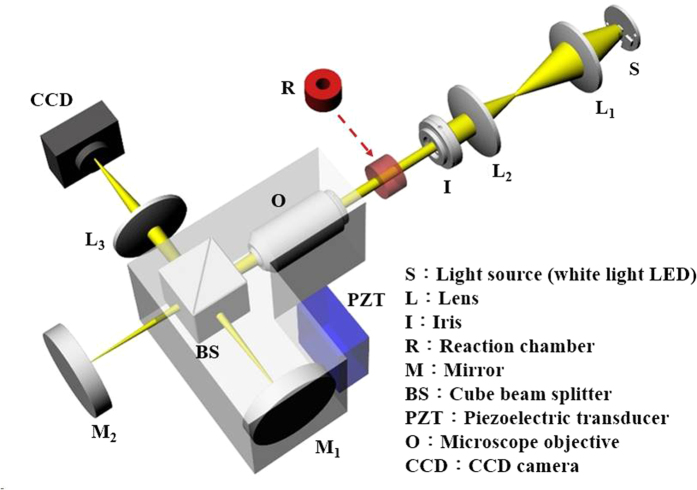
Schematic diagram of the WLSI-based spectrometer.

The setup of the WLSI-based spectrometer, based on a typical Michelson interferometer, as illustrated in Fig. 1, comprised a 5X Michelson interference objective (NA = 0.13) and a collimated LED light source. A vertical scanner was enabled by a piezoelectric transducer (PZT) with a closed-loop control and drove a moving mirror over an 18 μm range. The correlograms were acquired using a low-noise CCD camera with 640 × 480 pixels.

For the WLSI-based spectrometer, the optical length of the objective arm was changed by the moving mirror along the vertical (z) direction via the PZT, and the resulting interference signal from the two arms of the interferometer is expressed as^1^


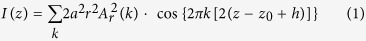


where *A*_*r*_ is the field amplitude of the white-light LED source and is a function of wavenumber *k*; *a* and *r* are the amplitude changes by the beam splitter and mirrors in the two arms, respectively; *z*_*0*_ is a constant to specify the position of the stationary mirror; and *h* represents the surface profile. The summation over the wavenumber *k* represents the superposition of interference signals over the entire spectrum in the light source. Then, using the inverse Fourier transformation, the Fourier spectrum of the interference signal is retrieved. Therefore, the Fourier amplitude is given by


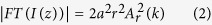


In fast Fourier transform notation, the wavenumber is expressed as *k*=*j/M*(2∆*z*); also, *M* is the total number of frames, Δ*z* is the frame interval, and *j* is the frame number (0 < *j* < *M*). Here the frame number and interval are 254 and 71 nm, respectively. A scan distance of 18 μm was used for the measurement. Therefore, the measured spectrum is 18 data points in the spectrum range of the LED source (506–665 nm).

**Figure 2:**
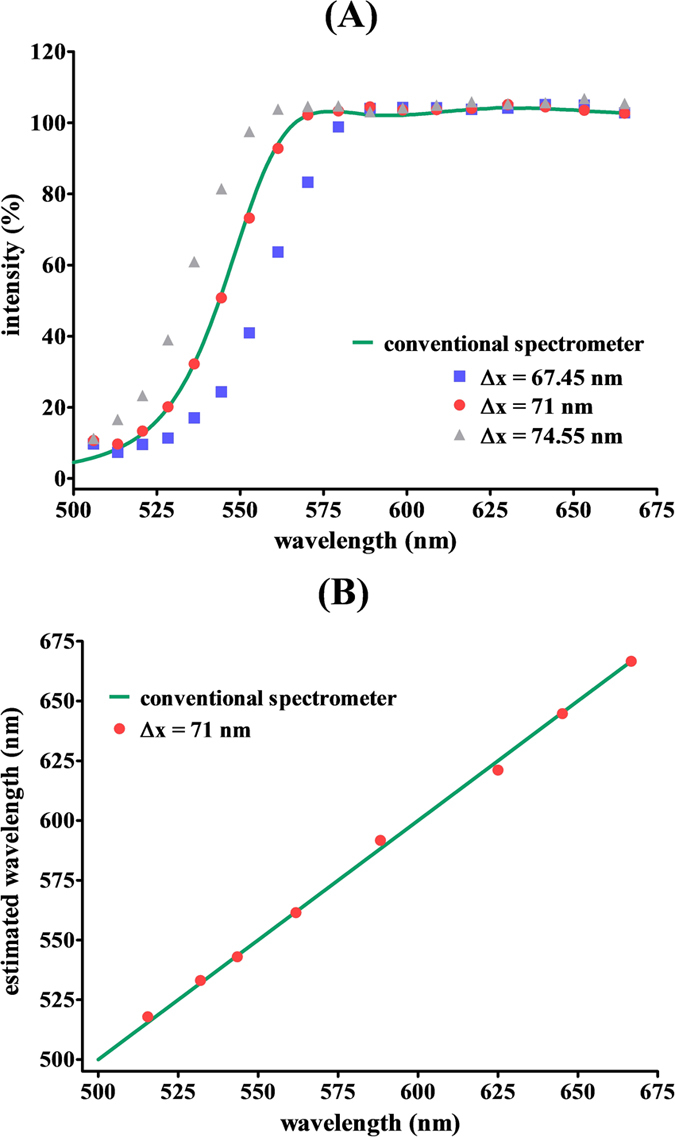
(**A**) Spectra of the edge filter measured using the WLSI-based spectrometer system at three frame intervals (Δz = 67.45, 71, and 71.55 nm) and the conventional spectrometer (solid curve). (**B**) Correlation (R^2^ = 0.9993) between the estimated wavelength and wavelength measured from the conventional UV-Vis spectrometer for the measurement using a frame interval of 71 nm.

First, the wavelength accuracy of the instruments should be verified because it is important for most applications. As mentioned above, the wavenumber k is inversely proportional to the frame interval Δz, which depends on the PZT actuator. Therefore, the actual wavelength accuracy is highly dependent on the frame interval Δz. Here we use an edge filter as a calibration standard to confirm the wavelength accuracy of the WLSI-based spectrometer. Three frame intervals (Δz = 67.45, 71, and 71.55 nm) were tested, and the results were compared to a reference spectrum that was measured by a conventional UV-Vis spectrometer, as shown in Fig. 2(A). It can be seen in Fig. 2(A) that the spectra were affected by the frame interval, and the result using a frame interval of 71 nm showed the best match to the reference spectrum. Meanwhile, we further evaluated the wavelength uncertainty for measurements with a frame interval of 71 nm. As shown in Fig. 2(B), the estimated wavelengths were observed to vary linearly with the wavelength measured from a conventional UV-Vis spectrometer with a coefficient of determination (R^2^) of 0.9993 and a mean percentage error of 0.4%. Thus, the proposed WLSI is sufficiently accurate to be used as a spectrometer. The frame interval was fixed at 71 nm in the following experiments.

### Physical performance testing of the WLSI-based spectrometer: refractive index variation measurements.

**Figure 3:**
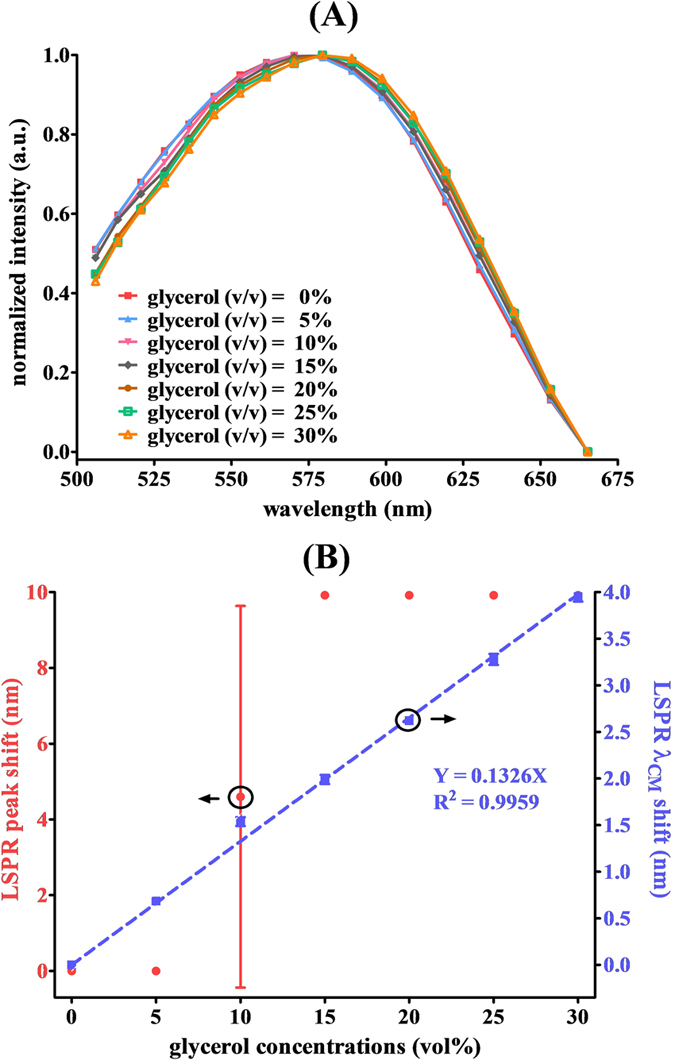
(**A**) The LSPR extinction spectra of SA-GNU in the presence of different concentrations of glycerol in water as measured by the WLSI-based spectrometer. (**B**) The LSPR peak shift (red solid circles) and the LSPR spectral centroid shifts (blue solid squares) of the SA-GNU extinction spectra as a function of the refractive index of the medium.

Measurement of the basic sensing characteristics of the WLSI-based spectrometer was executed using an LSPR extinction spectrum shift of streptavidin-conjugated gold nanourchins (SA-GNU) in various sensing media. Glycerol-water solutions with concentrations of 0, 5, 10, 15, 20, 25, and 30 vol% were treated as the sensing media, where vol% is the volume percent concentration. 0% glycerol-water solution is the control group. Each concentration was evaluated in at least triplicate analyses. The sample was inserted in front of the LED light source, and then one mirror was moved to occur interference. Finally, Fourier transform method was used to acquire the LSPR extinction spectrum. It can be clearly seen in Fig. 3(A) that the measured LSPR extinction spectra of SA-GNU showed a redshift with an increasing glycerol concentration as the refractive index of the medium increased. The LSPR peak shift of each spectrum was plotted versus the glycerol concentration, as seen in Fig. 3 (B, red solid circles). The results showed that the LSPR peak positions were the same at 0 and 5 vol% glycerol-water solutions, indicating that the spectral resolution was not high enough to resolve the LSPR peak shift. Additionally, the low spectral resolution also caused a significant measurement error at a glycerol concentration of 10 vol%. Consequently, the lowest detectable concentration of a glycerol-water solution was 15 vol% using the peak-shift method with the WLSI-based spectrometer. Thus, we adopted the spectral centroid algorithm to compensate for the poor spectral resolution. According to equation (3), the spectral centroid of the LSPR extinction spectrum of SA-GNU was calculated and plotted versus the glycerol concentration as seen in Fig. 3(B, blue solid square). The value of *λ*_*CM*_ varied linearly with the glycerol-water concentration over the range of 0 to 30 vol%, and the R^2^ is 0.9959. Based on the International Union of Pure and Applied Chemistry (IUPAC) definition, the theoretical LOD was estimated to be 0.82 vol%, corresponding to a refractive index variation of 1.2 × 10^−3^ refractive index units (RIU) relative to pure water.

### Refractive index variation measurements by the conventional UV-Vis spectrometer

**Figure 4:**
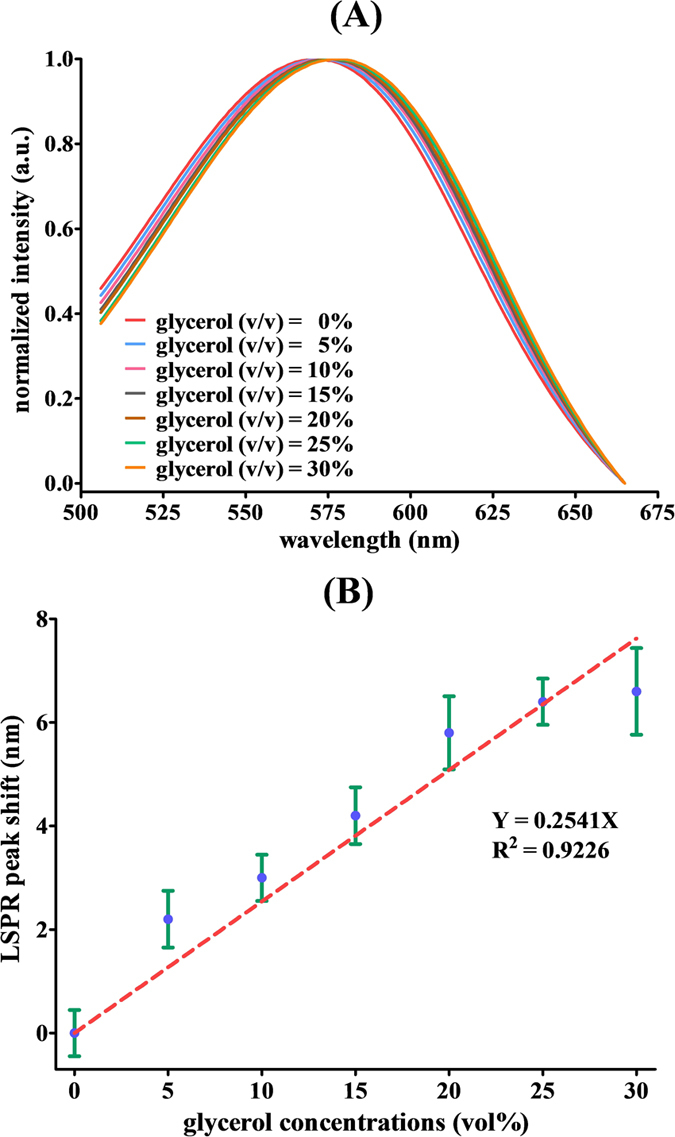
(**A**) The LSPR extinction spectra of SA-GNU in the presence of different concentrations of glycerol in water as measured by the conventional UV-Vis spectrometer. (**B**) The LSPR peak shift of the SA-GNU extinction spectra as a function of the refractive index of the medium.

To compare the performance of the proposed platform with the conventional method, a commercial UV-Vis spectrometer, Cary 300 Bio, was tested following the same experimental procedure. The measurements were carried out for glycerol-water solutions with concentrations of 0, 5, 10, 15, 20, 25, and 30 vol%. Each concentration was evaluated in at least triplicate analyses. The observed LSPR extinction spectra of SA-GNU were red-shifted with increasing glycerol concentrations as the refractive index of the medium increased, as shown in Fig. 4(A). Meanwhile, a linear relationship between the glycerol-water concentration and LSPR peak shift was obtained in Fig. 4(B), and the R^2^ is 0.9226. Therefore, the theoretical LOD was estimated to be 5.28 vol%, corresponding to a refractive index variation of 10.8 × 10^−3^ RIU relative to pure water. The LOD is almost an order of magnitude worse than that obtained by the proposed WLSI-based spectrometer using the spectral centroid algorithm. The results reveal that the spectral centroid method is able to significantly boost the performance of a spectrometer.

### Bioapplication: target strand detection by triggering SA-GNU aggregation

To demonstrate that the WLSI-based spectrometer can be used for biomedical applications, the developed spectrometer was employed to observe aggregation of GNUs for oligonucleotide detection. The SA-GNU surface was conjugated with biotinylated probes with sequences complementary to the target strand. After introducing target strands, SA-GNU quickly aggregated due to the interactions between the target strands and probes. The original concentration of the SA-GNU solution was 3.51 × 10^11^ nanoparticles/mL, and the binding capacity for 1 mg of SA-GNU to biotin is 2.33 nmol. The volume of the homemade reaction chamber was approximately 500 μL (16 × π × 10 mm^3^).

**Figure 5:**
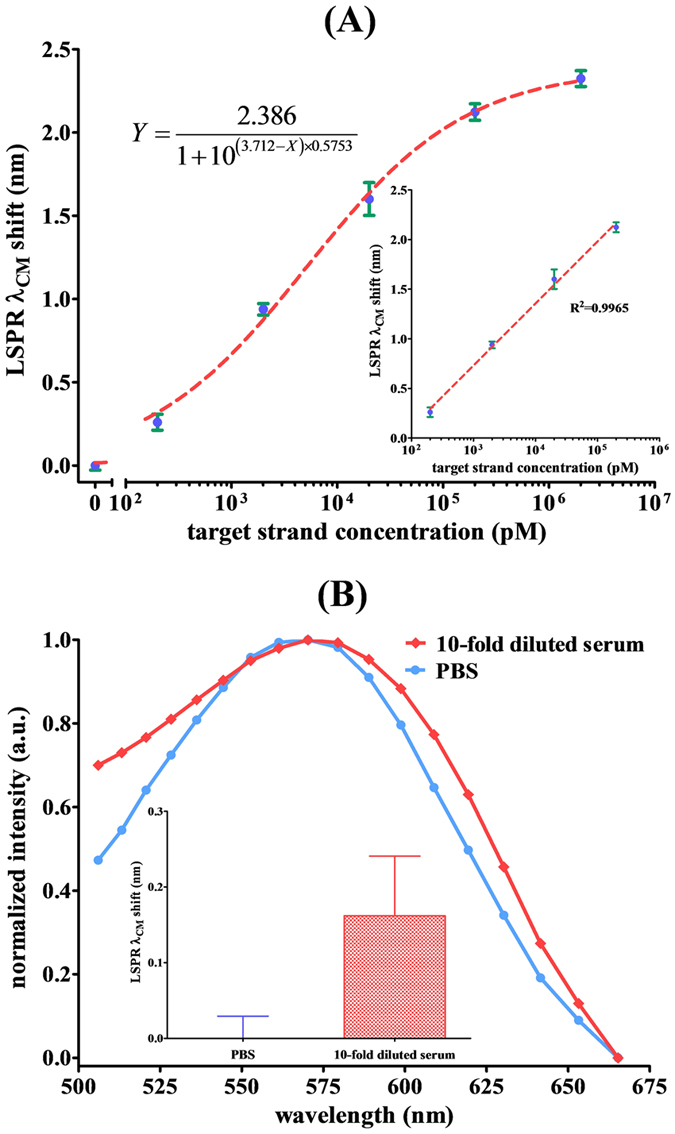
(**A**) Correlation (R^2^ = 0.9965) between the LSPR spectral centroid shift and the concentration of target strand, the rs242557 tau gene, over the range of 0 to 2 μM. Insert: linearity over the concentration range up to four orders of magnitude. (**B**) The LSPR extinction spectra of SA-GNU in PBS and in 10-fold diluted serum as measured by the WLSI-based spectrometer.

The diagram in Figure S1 (Supplementary Information) illustrates the formation of aggregated complexes, which were observed after incubating SA-GNU-probes with various target strand concentrations. When GNU aggregation is triggered by oligonucleotide hybridization, a redshift in the LSPR extinction spectrum provides a useful platform for biosensing because of the unique optical properties of the interparticle surface plasmon coupling^21^,^22^,^23^,^24^. To test the performance of the bioassay, the rs242557 tau gene, which is associated with Alzheimer’s and Parkinson’s diseases^19^,^20^,^25^, was selected as the target strand. A set of target concentrations was prepared by tenfold serial dilutions ranging from 2 μM to 200 pM: 2 μM, 200 nM, 20 nM, 2 nM, 200 pM. Meanwhile, zero concentration (blank) is the control group. Each concentration was evaluated in at least triplicate. Then, the correlation between the spectral centroid shifts and concentration of the target strand, the rs242557 tau gene, over the range of 0 to 2 μM, was plotted and shown in Fig. 5(A). The results were analyzed using a sigmoidal dose-response curve and exhibited linearity over the concentration range of 200 pM to 200 nM as well as a wide dynamic range up to four orders of magnitude (insert). The theoretical LOD was calculated as 15 pM based on both experimental data and the fit curve for this experimental design in accordance with the IUPAC definition.

To improve specificity, we used binary approach containing two individual probes complementary to different regions of the target. The sequences of the DNA strands used in this work, including two biotinylated probes and one target strand, were shown in Table S1 (Supplementary Information). The individual probes binding to a relatively short fragment of the target, makes the short duplexes extremely sensitive to single nucleotide substitutions^26^,^27^,^28^. When the short duplexes forming, gold nanoparticles aggregated, and a large shift in the spectral centroid of the LSPR extinction spectrum was caused only in the presence of the fully complementary targets, resulting in a remarkable increase in both the specificity and sensitivity. Following, the matrix background was evaluated through the 10-fold diluted serum response. The results, shown in Fig. 5(B), revealed a slight shift by ~0.16 nm in the LSPR spectral centroid for the 10-fold diluted serum. It corresponds to ~55 pM target DNA based on the correlation between the spectral centroid shifts and concentration of the target strand as presented in Fig. 5(A).

## Discussion

Currently, WLSI is widely used for surface profiling and thickness measurement of thin films by moving the reference mirror or target surface to occur interference. Thus, the specimen is usually placed at one of the two arms of the interferometer, and then an interferogram is generated when finding the zero optical path difference between the two arms. But, in the proposed WLSI-based spectrometer, the specimen is inserted in front of the LED light source, as illustrated in Fig. 1. Such optical design is more suitable for absorption-based measurements than traditional setup because the similar reflected intensities of the two arms would contribute to a high interference efficiency. This condition can easily be met in the proposed WLSI-based spectrometer.

Among various nanomaterials, GNPs have significant advantages over other nanomaterials in bioassays because of their unique optical properties, stability, activity, conductivity, biocompatibility, and surface chemistry^29^. In particular, GNPs exhibit LSPR at visible wavelengths, which depends on their sizes, shapes, dielectric constants, and surrounding environments^30^. Recently, non-spherical GNPs have attracted attention in the fields of chemistry and biology because the spiky uneven surface causes an enhanced electromagnetic field at the tips of the non-spherical GNP spikes, which is higher than for spherical GNPs^31^. Such non-spherical GNP would then be more sensitive to the refractive index changes in the vicinity^32^. This feature makes the non-spherical GNP such as GNU ideal for developing a sensitive LSPR-based detection assay. Moreover, assemblies of GNPs are especially interesting as the optical property is distinct from individual particles^33^. Mirkin’s group first reported the unique optical properties of an aggregate of GNPs, which induces interparticle plasmon coupling, resulting in a redshift in the LSPR spectrum^21^,^22^,^23^,^24^. The GNP aggregation causes a LSPR shift that is manifested as a useful spectrum-based biosensing platform^14^,^29^. Meanwhile, it belongs to a homogeneous binding assay, which is carried out in solution without sophisticated processes, such as separation, immobilization, or washing steps. Furthermore, in combination with different sized or shaped GNPs makes it possible to develop a multiplex assay for simultaneous monitoring of multiple biological samples.

In this work, a breadboard of WLSI integrated with the spectral centroid algorithm was proposed as a time-domain spectroscopic system. The temporal interferogram is a linear superposition of interference of each frequency of the system and can be Fourier-transformed to obtain the spectrum. The Fourier method belongs to a multiplex methodology which means that it measures the whole spectrum simultaneously without optical filters or dispersive elements^7^,^8^,^34^. As a consequence, there is no degradation of energy throughput, and a higher signal-to-noise ratio is observed than conventional scanning monochromator spectrometers. However, the use of WLSI as a spectrometer has not been reported to date because the spectral resolution of a Fourier transform-based spectrometer is restricted by the mirror travel distance. In this study, the moving mirror was driven over a traveling range of 18 μm by the PZT, corresponding to a spectral resolution of approximately 7–10 nanometers in the spectrum range of the LED light source, while the spectral resolution is far worse than that of commercial spectrometers. Hence, the proposed WLSI-based spectrometer exploits the spectral centroid method to compensate for the poor spectral resolution as a result of noise reduction by taking more data points (wavelengths) into account. Experimentally, the performance of the WLSI-based spectrometer was tested and compared with a conventional UV-Vis spectrometer by measuring the LSPR extinction spectrum shift related to the refractive index variation. The LOD of the WLSI-based spectrometer for refractive index variation measurements is successfully improved by almost an order of magnitude than the conventional method. Moreover, the bio-applicability of the proposed spectrometer was investigated using the rs242557 tau gene, an Alzheimer’s and Parkinson’s disease biomarker. In the near future, the proposed WLSI-based spectrometer has great potential as a high-throughput and multiplex biosensing platform because it could become a hyperspectral imaging system.

## Materials and Methods

### Chemicals and reagents

DNA strands, including two biotinylated probes and one target strand, were purchased from Integrated Device Technology (San Jose, CA, USA). The glycerol solution (refractive index = 1.4729)^35^ and phosphate buffered saline (PBS) tablets were purchased from Sigma (St. Louis, MO, USA). The SA-GNU were purchased from Cytodiagnostics (Ontario, Canada). The conventional UV-Vis spectrometer used in this study was a Cary 300 Bio (Varian Medical Systems, Palo Alto, CA, USA), and all solutions were prepared using Milli-Q (Millipore, Bedford, MA, USA) A-10 grade deionized water.

### Normalized intensity and spectral centroid of the extinction spectrum

The extinction spectra of the SA-GNUs were measured using the WLSI-based spectrometer and a conventional UV-Vis spectrometer. The spectra were normalized to a maximum height of 1 for convenient comparison of the two spectrometers.

An assortment of data processing algorithms has been used to determine the position of the spectral peaks. The spectral centroid method is a simple method that determines the geometric center of a spectrum. The spectral centroid of the LSPR extinction spectrum of a SA-GNU solution is calculated as follows:


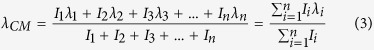


where *λ*_*CM*_ is the spectral centroid of the SA-GNU extinction spectrum; *I* is the normalized intensity of the SA-GNU extinction spectrum; *λ* is the wavelength; and *n* simply represents the pixels that are included in the calculation.

## Additional Information

**How to cite this article:** Li, M.-C. *et al*. An innovative application of time-domain spectroscopy on localized surface plasmon resonance sensing. *Sci. Rep.* **7**, 44555; doi: 10.1038/srep44555 (2017).

**Publisher's note:** Springer Nature remains neutral with regard to jurisdictional claims in published maps and institutional affiliations.

## Supplementary Material

Supplementary Information
