# Supplementary material for: An innovative application of time-domain spectroscopy on localized surface plasmon resonance sensing
Source: Sci Rep. 2017 Mar 10;7:44555. doi: 10.1038/srep44555 (PMC5345092; doi:10.1038/srep44555)
Supplement: Supplementary Information [file srep44555-s1.pdf]

An innovative application of time-domain spectroscopy on localized surface plasmon resonance sensing

Meng-Chi Li[1,+], Ying-Feng Chang[2,+], Huai-Yi Wang[1], Yu-Xen Lin[1], Chien-Cheng Kuo[1], Ja-an Annie Ho[2*], Cheng-Chung Lee[1*], Li-Chen Su[3*]

[1] Thin Film Technology Center / Department of Optics and Photonics, National Central University, Taoyuan 32001, Taiwan

[2] BioAnalytical Chemistry and Nanobiomedicine Laboratory, Department of Biochemical Science and Technology, National Taiwan University, Taipei 10617, Taiwan

[3] Department of Optoelectric Physics, Chinese Culture University, Taipei 11114, Taiwan

Author Contributions:

[+] these authors contributed equally to this work

* **Corresponding author.**

E-mail addresses: jaho@ntu.edu.tw (Ja-an Annie Ho), cclee@dop.ncu.edu.tw (Cheng-Chung Lee), slz@ulive.pccu.edu.tw (Li-Chen Su).

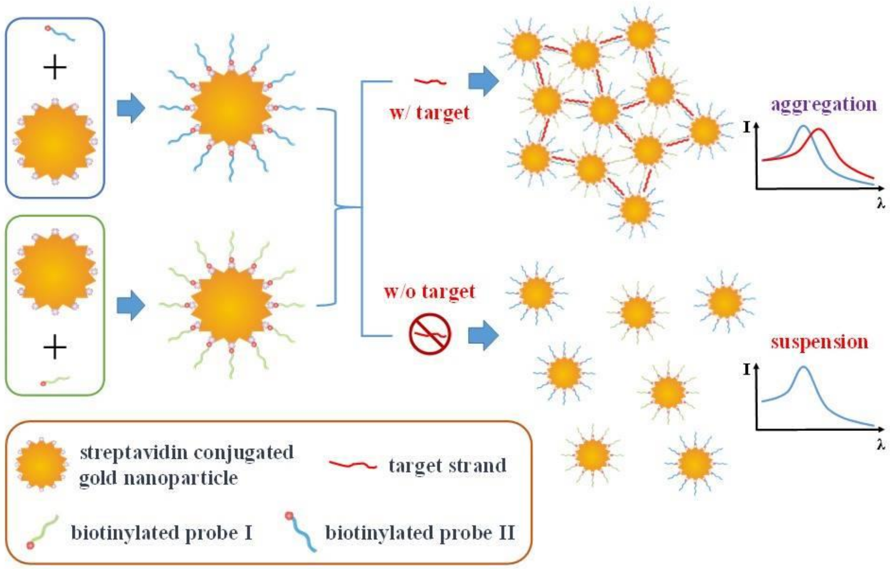

Figure S1. Schematic representation of the concept for generating aggregation signals. The SA-GNU-probes and oligonucleotide interconnections are not drawn to scale, and the number of oligomers per SA-GNU is likely much larger than depicted.

Table S1. Sequences of the DNA strands, including two biotinylated probes and one target strand.

Name	Sequence
**Target strand**	**5’ CCCAGGGTACACCAGGACACGGTTTT 3’**
**Biotinylated probe I**	**5’ GGTGTACCCTGGGTTTTT-biotin 3’**
**Biotinylated probe II**	**5’ biotin-TTTTTAAAACCGTGTCCT 3’**
